# Unveiling the Role of Mechanical Microenvironment in Hepatocellular Carcinoma: Molecular Mechanisms and Implications for Therapeutic Strategies

**DOI:** 10.7150/ijbs.102706

**Published:** 2024-09-30

**Authors:** Jiachen Hong, Jiongjie Yu, Damiano Buratto, Wei Chen, Ruhong Zhou, Sunbin Ling, Xiao Xu

**Affiliations:** 1 Hangzhou Normal University, Hangzhou, 311121, China.; 2 Zhejiang University School of Medicine, Hangzhou, Zhejiang, 310000, China.; 3 Institute of Quantitative Biology, and College of Life Sciences, Zhejiang University, 310027, Hangzhou, China.; 4 Department of Cell Biology, Zhejiang University School of Medicine, and Liangzhu Laboratory, Zhejiang University, Hangzhou, Zhejiang, China.; 5 The First Affiliated Hospital, School of Medicine, Zhejiang University, Hangzhou, 310058, China.; 6 Department of Hepatobiliary and Pancreatic Surgery and Minimally Invasive Surgery, Zhejiang Provincial People's Hospital (Affiliated People's Hospital), School of Clinical Medicine, Hangzhou Medical College, Hangzhou 314408, China.; 7 The Second Clinical College of Hangzhou Normal University, Hangzhou Normal University, Hangzhou, Zhejiang 311121, China.

**Keywords:** Hepatocellular carcinoma, Extracellular matrix, Mechanosensors, Mechanical microenvironment, Mechano-immunotherapy

## Abstract

Hepatocellular carcinoma (HCC) is the sixth most common cancer in the world and the third leading cause of cancer deaths globally. More than 80% of HCC patients have a background of fibrosis or cirrhosis, which leads to changes in physical factors in tumor microenvironment (TME), such as increased stiffness, solid stress, fluid stresses and structural alterations in the extracellular matrix (ECM). In the past, the focus of cancer research has predominantly been on genetic and biochemical factors in the TME, and the critical role of physical factors has often been overlooked. Recent discoveries suggest these unique physical signals are converted into biochemical signals through a mechanotransduction process that influences the biological behavior of tumor cells and stromal cells. This process facilitates the occurrence and progression of tumors. This review delves into the alterations in the mechanical microenvironment during the progression of liver fibrosis to HCC, the signaling pathways activated by physical signals, and the effects on both tumor and mesenchymal stromal cells. Furthermore, this paper summarizes and discusses the therapeutic options for targeting the mechanical aspects of the TME, offering valuable insights for future research into novel therapeutic avenues against HCC and other solid tumors.

## Introduction

Hepatocellular carcinoma (HCC) is one of the most common malignancies and a leading cause of cancer-related deaths worldwide^1^. More than 80% of HCC patients have a background of fibrosis or cirrhosis^2^. And advanced liver fibrosis or cirrhosis are risk factors of radical hepatectomy for late recurrence and can lead to multifocal recurrence of the remnant liver^3^. Some studies have confirmed the strong correlation between the fibrotic microenvironment in HCC, the malignant progression of the disease, and the patient's prognosis^2^. Compared with the median survival of patients with cirrhosis of 2.5 years, the median survival of patients without cirrhosis reached 5.8 years^4^.

Liver fibrosis induces both structural and functional changes in the liver, including intrahepatic microvascular malformations and remodeling of the extracellular matrix (ECM)^5^. Living cells are continuously subjected to mechanical stimulation from the surrounding ECM and neighboring cells. Cell membranes contain a variety of complex mechanosensors, such as integrins, ion channels, transient receptor potential (TRP) channels and piezo channels, which can convert external mechanical signals into biochemical signals recognized by cellular effectors, thereby activating various mechanotransduction signaling pathways^6^. Previous studies have focused on factors such as biochemistry, including cellular autophagy, apoptosis, and inflammatory pathways 7, 8. Recent research has highlighted the significance of physical signals in the tumor microenvironment (TME), such as degradation, cross-linking, and physical remodeling of the extracellular matrix, in modulating cellular behaviors^9^. During liver fibrosis and HCC, significant mechanical changes, such as alterations in tissue stiffness, shear flow and hydrostatic pressure, occur in the hepatic interstitial space and sinusoids^10^. Tumor cells can receive mechanical signals from their microenvironment and respond through mechanotransduction pathways, regulating their biological behavior 11-13. Additionally, alterations in the microenvironment can affect the peritumor vasculature, resulting in immune-poor zones that impede the effectiveness of clinical treatments and make drug delivery difficult^14^-^16^. Tissue fibrosis and ECM sclerosis are associated with the progression of HCC and other solid tumors, including pancreatic ductal adenocarcinoma (PDAC) and breast cancer^17^, ^18^. This review introduces the alteration of mechanical signals during the progression of liver fibrosis to HCC, the biobehavioral effects of mechanical signals on tumor cells and stromal cells, and summarizes the therapeutic strategies, challenge and future perspectives for targeting the mechanical microenvironment, which will provide insights for further exploration of new therapeutic targets.

## Mechanical microenvironmental components of HCC

Rapid tumor growth disrupts the structure and function of surrounding tissues, leading to unique physical signals of the TME, such as increased stiffness, elevated solid stress and fluid stresses, and structural alterations in the ECM^19^.

During liver fibrosis, activated hepatic stellate cells (HSCs) transform into myofibroblasts, which leads to the secretion and deposition of ECM components (fibrillar proteins, glycosaminoglycans, proteoglycans and mucins)^20^, ultimately resulting in matrix stiffening^21^. Typically, the elastic modulus is less than 6 kilopascals (kPa) for a healthy liver, 6-8 kPa for mild to moderate hepatic fibrosis, 8-12.5 kPa for severe hepatic fibrosis, and greater than 12.5 kPa for a cirrhotic liver^10^. Fibrosis-4 (FIB-4) is currently one of noninvasive blood-based serum tests proposed for fibrosis screening^22^. A high FIB-4 index and increased liver stiffness are positively associated with the risk of HCC development^23^, ^24^. The stiffness of HCC tissue is approximately ten times greater than that of normal liver tissue^25^. As the composition and stiffness of the ECM change, along with tumor growth, physical forces or pressures are exerted on tumor cells through both cell-cell and cell-matrix interactions. The physical forces exerted by fluid tumor components, known as liquid pressure, result in solid stress from growth and surrounding tissues. This can lead to the collapse or damage of blood and lymphatic vessels in and around a tumor, ultimately causing an increase in interstitial hydraulic pressure within the tumor 26.

Increased solid stress during tumor growth can cause the accumulation of radial and circumferential stresses in the tissue, leading to the deformation of cells and vasculature. The increasing solid stress is due to the combination of leaky vasculature and an imperfect drainage system, which results in the flow of fluid from the tumor through the tumor periphery^27^. Fluid stresses encompass microvascular and interstitial fluid pressure (IFP), and the shear stress exerted by blood and lymphatic flow on the vessel wall, as well as by interstitial flow on cancer and stromal cells and ECM^28^. Vascular shear stress is defined as the frictional force applied by blood or interstitial fluid flowing on the surface of hepatic cells. Structural damage to the liver caused by fibrosis, scarring and nodule formation, angiogenesis, and vascular occlusion increases resistance to blood flow within the liver^29^.

These attributes are closely interconnected, and any alteration in one attribute can trigger a positive feedback loop, potentially activating or intensifying the activity of other indicators 27. Stromal stiffness, IFP and interstitial shear stress were more elevated in the HCC tumor microenvironment compared to liver fibrosis^10^. In recent years, it has been discovered that unique physical signals in tumors affect cells as profoundly as chemical signals, with major implications for facilitating tumor progression and treatment, as we will develop below.

## Impact of mechanics signals on tumor cells behavior

**Figure 1:**
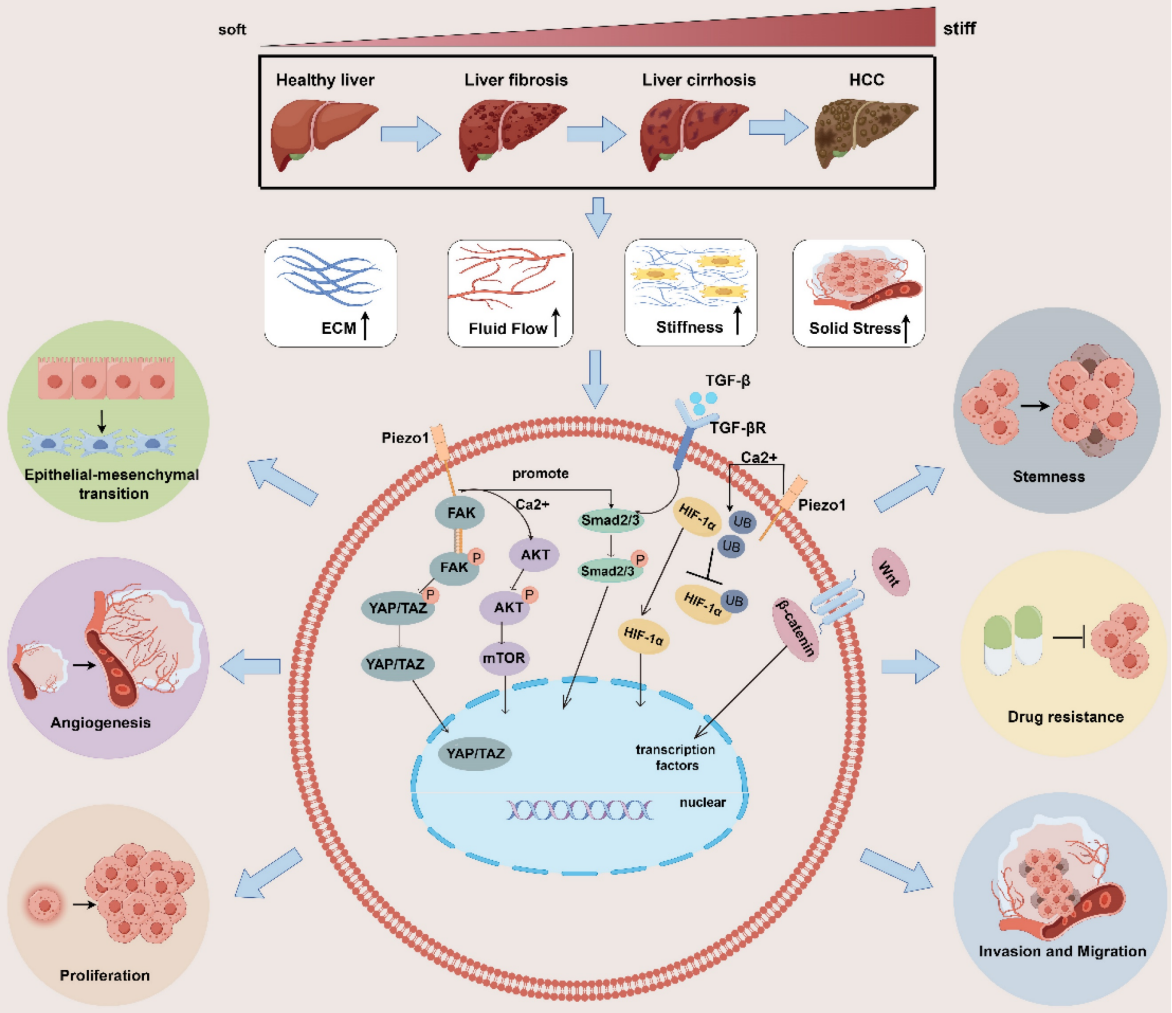
** Impact of mechanical microenvironmental alterations from healthy liver to fibrosis/cirrhosis and tumorigenesis on tumor behavior.** The progression of liver fibrosis to hepatocellular carcinoma leads to the development of a distinct mechanical microenvironment within the tumor. This microenvironment is characterized by increased stiffness of the surrounding stromal tissue, elevated solid stress, heightened interstitial fluid pressure, and altered structure of the ECM. These physical signals activate multiple signaling pathways, such as the HIF-α, YAP/TAZ, AKT/mTOR, and TGF-β/Smad2/3, Wnt/β-catenin pathways. The activation of these pathways promotes various behaviors in tumor cells, including proliferation, invasion and migration, epithelial-mesenchymal transition, angiogenesis, stemness, drug resistance, immune evasion and others. ECM: extracellular matrix; YAP: yes-associated protein;

Tumor cells and stromal cells can convert these mechanical signals into biochemical signals via mechanosensory or mechanotransporters^30^ and activate multiple pathways at the molecular level 31-33, thereby regulating tumor cell proliferation, invasion and migration, epithelial-mesenchymal transition(EMT), angiogenesis, stemness, drug resistance, immune evasion and others. In the following, we summarize and review the effects of these mechanical signals on tumor behavior (Figure 1).

### Enhancing proliferation

Mechanical signals generated by matrix stiffness can induce autophagy in fibroblasts or hepatic stellate cells via integrins and FAK, regulating the expression of the AMPKα protein, which promotes the growth of neighboring cancer cells^34^. Moreover, the stiffness generated by the ECM then activates the RhoA-Akt-P300 axis to promote the differentiation of neighboring hepatic stellate cells into myoblasts, promoting metastatic hepatocellular carcinoma growth^35^. The Hippo signaling pathway is crucial for tumor progression and pathogenesis because it connects stiffness and mechanotransduction to tumor progression. Yes-associated protein (YAP) is significant downstream signaling factor in the Hippo signaling pathway^36^. Apart from its role in promoting liver fibrosis and HCC through its interaction with the transcription enhancement associated domain transcription factor, YAP/TAZ is also widely acknowledged as a mechanical sensor capable of responding to various external physical stimuli 37, 38. Mechanical signals from rigid substrates are transmitted to cells through pathways such as focal adhesion → pFAK → cytoskeletal tension, ultimately inducing YAP/TAZ dephosphorylation^39^. The overexpression of the activated form of TAZ combined with NRAS (G12V) mutation promotes liver tumor formation but to a lesser extent than does the overexpression of activated YAP^40^. Moreover, elevated matrix stiffness does not affect YAP expression but decreases the proportion of phosphorylated YAP, promotes YAP nuclear translocation, and regulates gene transcription^41^. Animal experiments have revealed that drugs inducing dephosphorylation of YAP/YAZ can enhance hepatocyte proliferation and liver regeneration after partial hepatectomy in mice^42^, ^43^. In HCC, the sustained increase in YAP/TAZ activity not only initiates hepatic tumorigenesis^44^, ^45^, including promoting cell proliferation, inducing tumor cell tissue invasion, and maintaining cancer stem cells (CSCs)^46^, but also inhibits hepatocytes apoptosis^47^. In patients with Type 2 diabetes mellitus/non-alcoholic steatohepatitis, extracellular advanced glycation end products promote changes in collagen structure and increase ECM viscoelasticity, thereby driving the progression of HCC through activation of the integrin-β1-tensin-1-YAP mechanotransductive pathway^48^.

### Facilitating migration

Analysis of genome-wide RNA-Seq data from tumor and nontumor cirrhotic tissues of HCC patients revealed highly enriched gene signatures associated with extracellular matrix, fibrosis, and the EMT 49. Several factors contribute to the EMT in tumor cells, GSEA of HCC tissues revealed that in a Piezo1-high group, TGF-β signaling and the EMT are enriched; TGF-β can promote the EMT by regulating the expression of Snail transcription factors in the microenvironment; and Piezo1 promotes the proliferation, invasion and migration of HCC cells through Smad2/3, the canonical TGF-β signaling pathway^50^, ^51^. In addition, the upregulation of Piezo1 leads to the opening of ion channels, mediating an increase in Ca2+ levels. Subsequently, Ca2+ promotes the phosphorylation of Akt, which activates the downstream mTOR signaling pathway and promotes the assembly of cyclin D1-CDK4 complexes, which ultimately leads to the proliferation and migration of cancer cells^52^. Furthermore, in fibrotic hepatocellular carcinoma, increased stiffness of the ECM also promotes exosome secretion in a YAP/TAZ pathway-dependent manner, which indirectly promotes tumor cell motility and migration^53^.

### Stimulating angiogenesis

Angiogenesis provides essential nutrients for the growth and proliferation of HCC cells. Elevated solid stress in the tumor microenvironment can trigger the nuclear translocation of the transcription factor RUNX2 via Piezo1 activation. This, in turn, activates the Wnt/ β-catenin signaling pathway^54^, leading to the upregulation of COL4A1 and LAMC1 expression. These proteins, major constituents of the angiogenic and cancer cell ECM basement membrane, are upregulated in remodeling^55^. Additionally, excess ECM increases protein kinase C and splicing factor levels in endothelial cells to stimulate tumor angiogenesis^56^, ^57^. Stromal sclerosis upregulates the expression of vascular endothelial growth factor (VEGF) in HCC cells and vascular endothelial cells, suggesting that stromal sclerosis leads to tumor angiogenesis. Stiff stroma induces bone-bridging protein expression through the integrin β1/GSK-3β/β-catenin signaling pathway and may accelerate HCC progression 58.

### Inducing EMT

Cells can modify their migratory, invasive, and attachment capabilities through the EMT process, thereby evading drugs and the immune system^59^, ^60^. Experiments have shown that increased stromal stiffness significantly enhances the malignancy of HCC cells. This condition independently triggers EMT and activates three signaling pathways clustered on Snail expression, which collectively contribute to the stiffness-mediated EMT effect. In addition, key molecules required for stiffness-induced EMT exhibit high expression levels in tumor tissues from patients with HCC and elevated hepatic stiffness. Such conditions are associated with poor tumor differentiation and a high recurrence rate^61^. When HCC cells were grown in cirrhotic gel and hepatic fibrosis gel, cells grown in cirrhotic gel showed faster proliferation, larger tumor nodules and higher levels of EMT marker expression compared to those in the fibrosis gel 62.

Increased tissue stiffness leads to the nuclear accumulation of YAP/TAZ, altering the expression of E/N cadherin and vimentin. Meanwhile, guanine nucleotide exchange factor-H1 may facilitate the biomechanical signals from β1 integrin to YAP through shear stress-induced remodeling of F-actin, which would then encourage YAP's nuclear translocation^63^. This process induces EMT transformation, thereby promoting tumor invasion and metastasis^64^, ^65^. Increased stromal stiffness enhances TGF-β1-induced Smad signaling in HCC cells, which signals is considered the major EMT promoter and plays an important role in supporting tumor growth^66^.

### Promoting stemness

Biomechanical forces promote tumor stemness through integrin-cytoskeletal prestress-AIRE signals while mediating the quiescence of stem-like tumor cells through DDR/STAT1/P27 signaling 67. In stemness regulation of HCC cells, matrix stiffening may promote β1 integrin/Akt/mechanistic target of rapamycin (mTOR)/sex-determining region Y box 2 (SOX2) signaling^10^. Moreover, the stiffness of the HCC microenvironment varies and can directly affect the expression of stem-like characteristics in cells through mechanotransduction. Experiments with SMMC-7721 cells cultured on stiff polyacrylamide hydrogels have shown an increase in the expression of genes associated with stemness^68^. With increasing substrate stiffness, HCC cells exhibited a higher proportion of cells expressing CD133(+)/EpCAM(+), alongside with elevated expression levels of CD133, EpCAM, Nanog, and SOX2, indicating a link between stiffness and key signaling pathways involved in cell growth and survival.

### Augmenting drug resistance

The tumor stroma can limit the access of therapeutic agents to target tissues through three pathways: fibrosis, high interstitial pressure, and degradation of drugs by stromal enzymes^69^. A dense ECM reduces the density of blood vessels and causes them to be buried within the stroma, creating a strong barrier that drugs cannot perfuse. In addition, cancer cells can strongly adhere to ECM proteins to evade chemotherapy, a process known as cell adhesion-mediated drug resistance. Mesenchymal pressure is greater in malignant tissue than in nonmalignant tissues, and this difference can affect drug diffusion and transport^69^.

A stiff ECM in HCC can act as a physical barrier to the migration and infiltration of NK cells and CD8+ T cells into tumors, thereby disrupting the process of immune recognition and tumor cell destruction^70^. In the context of mechanical signaling, cells in HCC and ovarian carcinoma cells within a stiffer mechanical microenvironment exhibit reduced sensitivity to platinum-based treatment 66, 71. In a study of reduced sensitivity to sorafenib in HCC, increased matrix stiffness was found to promote the activation of integrin-JNK signaling^72^. Recent clinical studies indicate that patients with colorectal cancer and liver metastases have higher liver tumor tissue stiffness compared to those without metastases. Strategies targeting the renin-angiotensin system have been shown to mitigate fibroblast contraction and ECM deposition, reducing liver stiffness and increasing the anti-angiogenic effect of treatments like bevacizumab^73^. The three pathways of targeting the tumor stroma to promote drug resistance can help to improve drug resistance in tumors, and both immune and targeted therapies developed along these lines are currently achieving some results, which we will address in Chapter Five.

## Influence of Mechanical microenvironment on stromal cells

The HCC microenvironment has a variety of stromal cell components in addition to tumor cells, including immune cells, endothelial cells, and cancer-associated fibroblasts (CAFs), which promote tumor progression^74^, ^75^. In solid tumors, mesenchymal components interact with tumor cells to influence tumor behavior, and tumor cells can also form a microenvironment that supports tumor cell growth by altering the surrounding stroma. The tumor mesenchyme is also involved in tumorigenesis, progression, and treatment resistance and influences many features of cancer. Here, we focus on the microenvironmental changes in fibrotic tumors and provide a summary of the effects of mechanical signals on stromal cells and cell‒cell interactions.

### Interactions with immune cells

**Figure 2:**
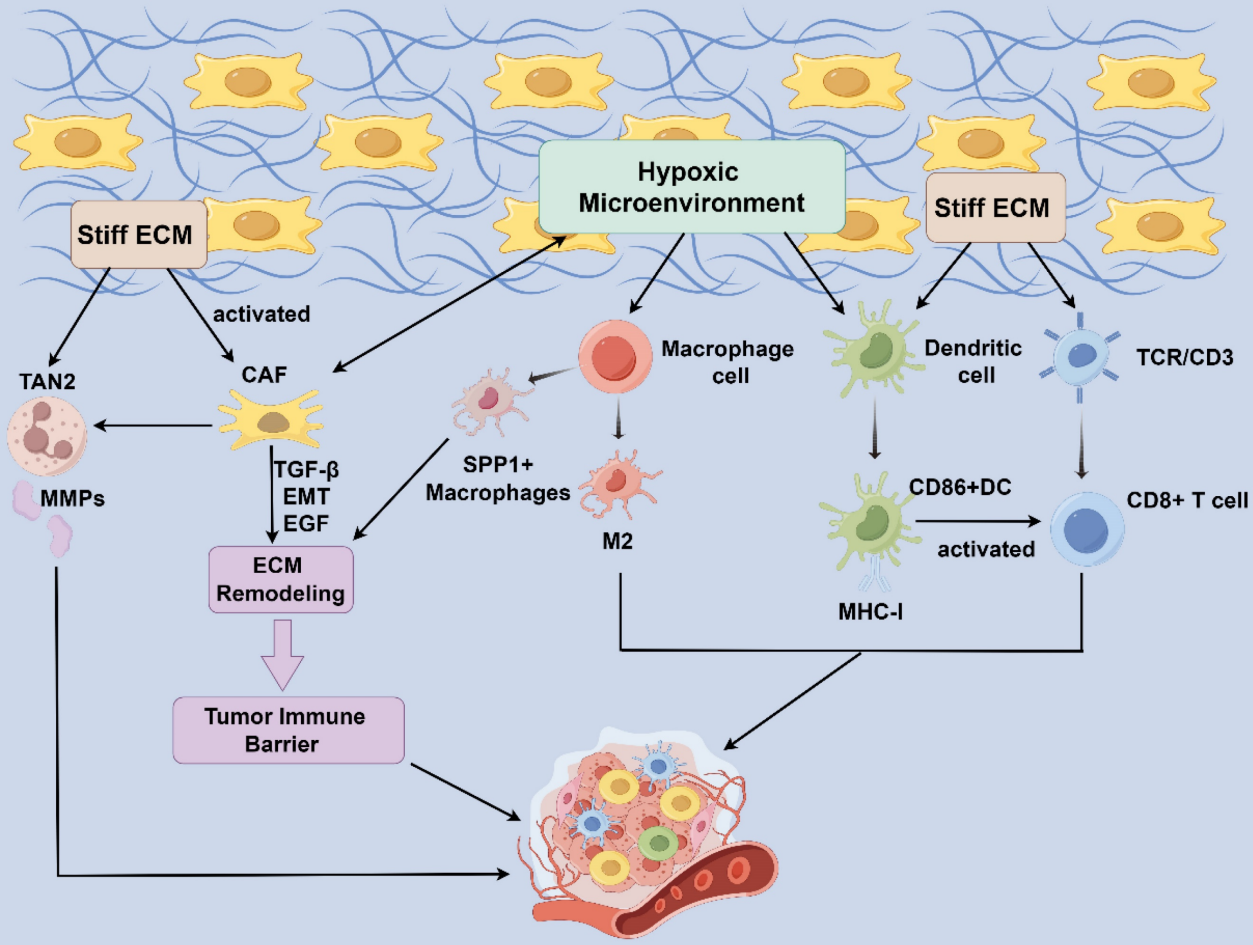
** Influence of Mechanical microenvironment on stromal cells.** Compression of peripheral blood vessels by increasing fibrous tissues in the HCC TME creates a local hypoxic microenvironment. CAF produces paracrine growth factors, protein hydrolases, and ECM components. SPP1+ macrophage interact with CAF to stimulate ECM remodeling and promote the formation of tumor immune barrier structures. CAFs increased TAN2 intratumoral infiltration while TAN2 released matrix metalloproteinases (MMPs) in turn induce the CAF phenotype, developing a positive feedback loop. Connective tissue proliferation accompanied by excessive deposition of ECM generates high stresses within the tumor, leading to hypoxia within the TME and a positive feedback loop. Under shear stress (pressure, matrix stiffness, arterial transmural pressure, etc.), the expression of DC activation markers MHC-I and CD86 increases, presenting a shape best suited for T-cell stimulation and activating the immune response. HCC: hepatocellular carcinoma; TME: tumor microenvironment; ECM: extracellular matrix; CAFs: cancer-associated fibroblasts; TANs, Tumor-associated neutrophils; TAN1: anti-tumor phenotype TANs; TAN2, pro-tumor phenotype TANs; MMPs: matrix metalloproteinase.

The ability of immune cells to sense and respond to the physical environment is crucial for their function. Recent evidence suggests that, mechanotransduction allows cells to convert external biophysical stimuli into intracellular biochemical signals^76^. In both physiological and pathological states, cells are influenced by the mechanical properties of their extracellular environment, including matrix stiffness, stretching, shearing, elasticity, and shape. These mechanical stimuli are interpreted by cells through mechanotransduction, leading to protein conformational changes, the activation of intracellular signaling pathways, and the regulation of gene expression, ultimately driving cellular functions^76^-^78^. These biophysical signals play a crucial role in regulating various immune cell functions, such as leukocyte extravasation, macrophage polarization, T cell selection, and T cell activation^78^ (Figure 2). This insight into the mechanistic underpinnings of immune cell regulation within the TME underscores the potential for novel therapeutic strategies targeting these biophysical pathways.

#### Macrophages

Tumor associated microphages (TAMs) are the most common infiltrating immune cells in tumor tissues. Classically activated M1 macrophages produce reactive oxygen intermediates, reactive nitrogen intermediates, and tumor necrosis factor-alpha to limit tumor growth. In contrast, M2 macrophages promote tumor growth and metastasis by secreting matrix-degrading enzymes, angiogenic factors, immunosuppressive cytokines, and chemokines^79^, ^80^. Macrophages can sense and respond to signals from the microenvironment to accomplish these functions. The secretion of Wnt ligands by HCC cells plays a role in promoting TAM polarization toward the M2 phenotype through the Wnt/β-catenin signaling pathway, ultimately leading to increased hepatocellular carcinoma proliferation, invasive metastasis, and immunosuppression^81^.

Compression of peripheral blood vessels by increasing fibrous tissues in the HCC TME creates a local hypoxic microenvironment that promotes SPP1 expression, whereas SPP1+ macrophages interact with CAFs to stimulate extracellular mesenchymal remodeling and promote the formation of tumor immune barrier (TIB) structures, thereby limiting immune infiltration in the core of tumors^82^. Physiologically relevant forces caused by interstitial flow and hydrostatic pressure may modulate macrophage polarization^83^, ^84^. In most solid tumors, TAMs within tumor tissue experience greater interstitial fluid flow than those within normal tissue. Interstitial flow polarizes mouse bone marrow-derived macrophages to an M2-like phenotype via integrin/Src-mediated signaling^85^ and promotes a mesenchymal phenotype in tumor cells^79^. These studies suggest that mechanical microenvironment modification promotes TAM polarization and HCC development through immunosuppression, highlighting TAM targeting as a potential therapeutic strategy for cancer therapy.

#### T cells

Increasing evidence shows that biomechanical cues are critical for T cell function and enhance T cell sensitivity to biochemical signals^86^-^88^. Traditionally, TCR signaling has been viewed as a purely biochemical process initiated upon recognition of pMHCs, but recent studies have shown that mechanical forces can mediate T cell signaling through the TCR/CD3 complex and the costimulatory receptor CD28^89^, ^90^. When the mechanosensor Piezo1 in T cells is blocked, it enhances their traction and intensifies cytotoxicity against tumor cells^91^. The presence of cytotoxic CD8+ lymphocytes in the TME is suggestive of a favorable prognosis and can also indicate the efficacy of drug therapy^92^. However, increased matrix stiffness inhibits the penetration of cytotoxic CD8+ T cells into tumor tissue^93^. T cell activation, proliferation, and migration are greater in 3D matrices with greater mechanical strength than in those with softer materials^94^. The expression of cytotoxic T cells activity markers is significantly downregulated, and the expression of regulatory T cells markers is upregulated in high-density stroma compared to low-density stroma, suggesting that the collagen density of the TME modulates the cytotoxic activity of tumor-infiltrating T cells to support the escape of tumor cells from immune destruction^95^. Recent studies have shown that many Tregs are distributed at sites of fibroplasia and that in the TME, Tregs exert immunosuppressive effects mainly through the inhibition of cell‒cell contact, the expression of surface molecules, and the secretion of cytokines.

#### Dendritic cells (DCs)

DCs are the most powerful specialized antigenic precursor cells, and their maturation leads to an increase in their cellular stiffness through cytoskeletal remodeling 87. Their main feature is the stimulation of the proliferation of primary T cell proliferation^96^. The fibroblast stroma and peritumor-associated ECM surrounding tumors impose physical constraints on DC infiltration, influencing DC maturation and mobility, which in turn affects T cell activity^73^. When an immune cell attaches or migrates to another cell or ECM and forms an immune synapse with an antigen-presenting cell, mechanical forces on the immune receptor occur 76, activating DCs, which then migrate to the site of inflammation in the draining lymph node 97. Exposure to shear stress, including pressure variation, matrix stiffness, and arterial transmural pressure, elevates the expression of DC activation markers, such as MHC-I and CD86. This adjustment optimizes their shape for efficient T-cell stimulation, thereby enhancing the immune response^98^.

#### Neutrophils

Tumor-associated neutrophils (TANs) are categorized into two populations: anti-tumor phenotype TANs (TAN1) and pro-tumor phenotype TANs (TAN2)^99^. They exhibit antimicrobial and inflammatory functions through phagocytosis, degranulation, the release of neutrophil extracellular traps (NETs), and antigen presentation^100^. The increase in neutrophils and NETs contributes to the tumor inflammatory response, thereby promoting the growth and progression of HCC. TAN2 primarily facilitates tumor growth *in vivo* by inducing angiogenesis and promoting cancer stem cells. It has demonstrated an increase in neutrophils in late-stage cirrhotic HCC, and NETs enrichment has been observed in HCC patients with high ECM deposition^101^. Additionally, a complex cellular crosstalk exists between CAFs, tumor cells, and TANs. It was reported that CAFs increased TAN2 intratumoral infiltration through the CLCF1/CNTFR signaling pathway, while matrix metalloproteinases released by TANs may in turn induce the CAFs phenotype, creating a positive feedback loop that accelerates HCC progression^102^. Currently, the research on neutrophils in the mechanical microenvironment is limited and needs to be further explored.

### CAFs

Under normal physiological conditions, normal tissue fibroblasts are in a quiescent state. During liver fibrosis, inflammation and activation of the innate immune system following hepatocellular injury lead to the activation of hepatic stellate cells, which gradually differentiate into CAFs 103. CAFs can remodel the ECM to provide a pathway for cancer cell metastasis, but the investigation of the mechanism is unclear, and one of the more dominant possibilities is that CAFs and cancer cells are guided to each other through mechanical interaction. CAFs can leverage N-calmodulin on the membrane and E-calmodulin on the membrane of cancer cells to adhere to each other, triggering the recruitment of β-catenin and exerting a physical force on cancer cells to pull them away from the tumor primary foci to facilitate the invasion, and at the same time, the cancer cells can leverage polarized CAFs to move away from the tumor to enhance the diffusion, and under the joint action of the two mechanisms, it ultimately facilitates the tumor metastasis and proliferation 104. In addition, with altered mechanical signaling, CAFs usually act in concert with immune cells, manifesting as fibrous tumors with an inflammatory phenotype, which further promotes fibrosis^105^-^107^ and ultimately promotes tumorigenesis, invasion, and drug resistance. Therefore, designing drugs against CAFs that block mechanical signaling activation can reduce their tumor-promoting effects.

## Therapeutic strategies for targeting the mechanical microenvironment

HCC as a complex three-dimensional structure, often exhibits different mechanical properties across its periphery and interior. The interior of HCC is subjected to compressive stresses mainly from tumor cells and sclerotic stroma, while the periphery of such tumors is subjected to tensile stresses from the mass and surrounding tissues^108^. Previous studies have shown that large amounts of collagen and hyaluronic acid (HA) accumulate inside tumors^109^, ^110^. These factors are crucial for the development of drug resistance, stemness, metastasis, the EMT, and various other tumor characteristics. Therefore, the inhibition of fiber contraction and the degradation of collagen and HA are expected to reduce mechanical stress in tumor tissues. Many therapeutic strategies have been investigated in this area, and basic and clinical trials are summarized in this chapter to provide new ideas for the treatment of HCC.

### Modulating the ECM

The ECM consists of proteins, proteoglycans, and glycosaminoglycans^111^. Disruption of the collagen arrangement is the main cause of structural abnormalities in the tumor ECM. A stiff ECM around a tumor attenuates the efficacy of chemotherapy and immunotherapy by preventing drug penetration and immune cell motility^112^. In recent years, the inhibition of ECM generation and hardening has become a new direction for the treatment of tumors. Therapeutic approaches include the following: 1. inhibiting the main components of the ECM such as collagen and HA (Table 1); 2. degrading the matrix and reducing its crosslinking properties (Table 1); 3. targeting the CAFs responsible for ECM hardening (Table 2).

HA, the simplest glycosaminoglycan and one of the main components of the ECM, may be a potential therapeutic target for reducing physical barriers to systemic therapy (Table 1). A study showed that when HA was inhibited, the expression of the CSCs markers CD44, CD133, and CD90 decreased accordingly^113^, suggesting that HA may be involved in the formation of CSCs. Some scholars have exploited this property to design HA-mediated Fe3O4 nanocubes, which were targeted to reverse CSCs through the binding of HA and CD44 to promote CSCs EMT, thereby inhibiting HCC proliferation and migration^114^. Using a lower dose of anti-VEGF antibody can reduce the stiffness and mechanical forces of tumor. HA depletion attenuates perfusion injury in mice model of metastatic colorectal cancer (mCRC) in liver and systemic chemotherapeutic response in patients^115^. In drug-resistant pancreatic cancer, the application of an HA-degrading drug (PEGPH20) normalizes IFP and enhances the delivery of cytotoxic drugs 116. Collagen disruption can facilitate the penetration of many conventional chemotherapeutic drugs and nanoparticles through the barrier of the hardened matrix in the TME for antitumor effects. In addition to inhibiting the production of matrix-associated proteins, degrading the cross-linking properties of the matrix has also been shown to hold therapeutic promise for treating tumors.

The lysyl oxidase (LOX) family (LOX, LOXL1, LOXL2, LOXL3) comprises copper-dependent monoamine oxidases in the ECM that catalyze the cross-linking of collagen and elastin. LOX inhibition significantly reduces the amount of collagen in the TME. For example, the LOX inhibitor β-aminopropionitrile (BAPN) inhibited the accumulation of cross-linked collagen during fibrotic progression in mice^117^. Losartan reduces LOX levels, inhibits cross-linking of type I collagen, and ultimately attenuates integrin-delivered cell signaling to inhibit tumor cell metastasis^118^. Collagen destruction can facilitate the penetration of many conventional chemotherapeutic agents and nanoparticles through the barrier of the hardened matrix in the TME. The indolyl tetramers PXS-5153A is LOXL2/LOXL3 inhibitor that has shown antifibrotic activity in preclinical models of hepatic and pulmonary metastases^119^. LOXL2 is barely expressed in healthy liver, and in liver fibrosis, inhibition of LOXL2 suppresses fibrosis progression^120^, ^121^. Simtuzumab is a humanized anti-LOXL2 IgG4 monoclonal antibody; although the drug has shown promising preclinical and tolerable results, phase II studies of simtuzumab have shown no clinical benefit in patients with advanced fibrosis due to nonalcoholic steatohepatitis^122^. To date, the outlook for the use of simtuzumab in cancer treatment remains poor.

Numerous therapeutic approaches have been utilized due to the shared mechanisms between tissue fibrosis and tumor fibrosis. The dysregulation of matrix protein production and remodeling in the TME is primarily attributed to the abnormal proliferation and differentiation of tumor fibroblasts, also known as CAFs. To date, therapeutic approaches for CAFs have focused on 3 main points: 1) the elimination of tumorigenic CAFs subsets; 2) the reversal of activated CAFs and tumor mesenchyme; and 3) the inhibition of CAFs activation signals. The following table summarizes the currently available CAF-targeted therapies and other antifibrotic therapies (Table 2).

α-SMA and FAP-positive CAF subpopulations play important roles in tumor development. In an A549 lung cancer model, FAP-specific T cells reduced the number of FAP-positive stromal cells and inhibited tumor growth^123^-^125^. Vaccines targeting FAP antigens can eliminate FAP-positive CAF subpopulations, leading to a decrease in collagen density in the ECM and an increase in chemotherapeutic drug uptake^126^,^127^. However, α-SMA and FAP are not CAF-specific cellular markers, making such subpopulations difficult to target with drugs alone. The selective elimination of the CAF subpopulation is ineffective, and therefore, the reversal of activated CAFs and tumor stroma and the inhibition of CAF activation signals have gradually attracted the attention of researchers. Retinoic acid and all-trans retinoic acid induce quiescence and reduce the motility of pancreatic stellate cells, reduce fibrosis, increase drug penetration, and decrease the proliferation and increase the apoptosis of surrounding pancreatic cancer cells 128,129. The vitamin D receptor ligand calcipotriol reduces inflammation and fibrosis in the pancreatic cancer tumor stroma, leading to induced stromal remodeling^130^. In a mouse model of PDAC, Halofuginone reduced fibroblast activation, decreased key extracellular mesenchymal elements that drive stromal resistance, improved drug delivery efficiency, and increased the infiltration of inflammatory macrophages and cytotoxic T cells 131. The activation signals of CAFs mainly include TGF-β and connective tissue growth factor. When the TGF-β pathway is inhibited by chlorthalidone, collagen I and HA deposition can be reduced, which can enhance vascular integrity and improve drug delivery to tumors^132^. However, more studies are still needed to determine which TGF-β signaling components can serve as common targets for antitumor therapy^133^. Indeed, due to the bidirectional crosstalk between CAFs and cancer cells, any therapeutic strategy targeting CAFs or cancer cells may not be able to achieve optimal efficacy, and more studies are needed to further explore this topic.

### Targeting mechanosensors or mechanotransducers

Previous studies have demonstrated that several mechanosensors and mechanotransducers are amenable to being targets for drug action. Inhibitors of integrins, Piezo1, TRPV4, and YAP have been developed. Many of these inhibitors have shown anticancer activity in preclinical studies (Table 3). The results of an animal study showed that integrin inhibitors could inhibit angiogenesis and proliferation in rats with HCC. Among the integrin family, αvβ3 has become an important target for tumor diagnosis and antitumor drug research due to its high expression in tumor neovascularization, and two preclinical experiments (Table 3) have demonstrated its potential in the treatment of solid tumors. In addition to αvβ3, other integrin isoforms have shown therapeutic potential; for example, a study constructed a monoclonal antibody targeting the CD151/a6β1 junction, which impeded the progression of HCC. In addition, the β8 subunit of integrins has been shown to contribute to resistance to lenvatinib in liver cancer patients 134.

In addition to the integrin family, good progress has been made in clinical or basic research on other mechanosensors or transducers. Several inhibitors, such as those targeting FAK, have shown substantial evidence in preclinical trials that targeting FAK as a clinical combination strategy can reverse the failure of chemotherapy or targeted therapy and thus improve the efficacy of immunotherapy for solid tumors 135. In addition, basic research on other drugs developed against YAP, TRPV4, Piezo1, and Rho kinase has yielded results that indicate treatment efficacy^136^-^141^.

### Advancements in mechano-immunotherapy

Recent studies in molecular medicine have revealed that mechanical forces also play a role in regulating the biological behavior of various types of cells, especially immune cells; this increased understanding of the mechanobiology of immune cells provides a good basis for developing new therapeutic strategies. Mechanical immunoengineering, as a nascent field, utilize biomechanical factors to modulate immune cell differentiation, proliferation, effector functions, etc., for diagnosis or therapy. It provides an additional dimension, complementary to traditional modulation of biochemical factors, to tailor immune cell responses and enhance therapeutic efficacy^142^.

#### Promoting of tumor cell stiffening

Tumor tissues are usually more rigid than normal tissues. Nevertheless, cancer cells exhibit softer characteristics than normal cells, which, independent of other biochemical features of cancer cells, act as mechanical "immune checkpoints" that induce the immune evasion of tumor cells 143-145. Emerging research has focused on strengthening cancer cells and sensitizing them to immune surveillance. The depletion of membrane cholesterol in cancer cells leads to increased stiffness, which in turn enhances T cell activity and cytotoxicity by overcoming mechanical immune checkpoints 145, 146. Using methyl-β-cyclodextrin to deplete membrane cholesterol and consequently stiffen tumor cells, this treatment also increased the susceptibility of cancer cells to T cell-mediated killing both *in vitro* and *in vivo*^145^. Enhanced cancer cell stiffness also facilitates macrophage phagocytosis and sensitization to current immunotherapies^145^. The inhibition of cholesterol biosynthesis is a viable and promising therapeutic strategy for the treatment of cancer, and the modulation of cholesterol levels at the plasma membrane may offer a novel alternative approach.

#### Enhancing immunotherapeutic efficacy

A stiff ECM not only hinders the penetration of conventional drugs but also blocks the penetration of immune cells 95. In response, drugs have been developed to improve the ability of immune cells to cross the stromal barrier, and some progress has been made.

A peptide ligand known as CSG can bind to the laminin-nitrogen complex in the ECM of mouse and human tumors and selectively deliver fusion proteins into the tumor ECM and subsequently trigger the infiltration of potent immune cells while degrading the rigid tumor extracellular matrix, improving nanomedicine uptake and prolonging the survival of tumor-bearing mice 147. Some ultrasmall silica nanoparticles can directly bind to TCR-CD3 complexes and activate T cells 148. The ultrasmall nanoparticles have good prospects for clinical translation because they avoid liver accumulation and are rapidly cleared by the kidney. Magnetic nano complexes effectively attach to NK cells and promote the generation and secretion of NK cell lysate particles, which improves the efficacy of NK cells against HCC 149.

Recent studies have reported the use of ultrasound to stimulate the Piezo1 ion channel, activating the dephosphorylation of the nuclear factor of T-cells and transferring the activated nuclear factor of T-cells to the nucleus of the cell. This process activates the transcription of the Chimeric antigen receptor (CAR) gene, a technique known as "remote-controlled mechanogenetics", which can effectively control CAR expression in T cells to enhance tumor cell recognition and eradication, leading to controlled cancer immunotherapy 150. Although the clinical potential of these emerging studies as cancer immunotherapies has yet to be fully realized, they underscore the promising interaction of mechano-immunoengineering and cancer treatment. As our understanding of these disciplines deepens, it is anticipated that more effective therapies will be proposed and implemented.

## Conclusion

The various types of mechanical signals in the HCC microenvironment constitute a complex and tightly regulated mechanoregulatory network through the activation of different signaling pathways, thus affecting the development of HCC. A better understanding of how tumor cells receive and integrate these mechanical signals is crucial for the development of new anticancer therapies.

Currently, there exist challenges for the application of therapy targeting mechanical microenvironmental in clinical. Related research is still at the preclinical and early clinical trial stages, with some still in the basic research phase. Future efforts should focus on advancing large-scale, multicenter, prospective clinical trials. Additionally, in clinical practice, technologies for objective and accurate assessment for mechanical microenvironment are currently limited and still relied on postoperative pathology. However, with the rapid development of artificial intelligence and radiomics, there may be new opportunities for non-invasive and precise preoperative evaluation of mechanical microenvironment.

Our review suggests that promising therapeutic strategies lie in identifying and targeting novel mechanical stress-related targets. These include mechanisms influenced by the ECM, cellular interaction within the tumor, and effects on intratumoral vascular tissue and immune cells, offering fresh perspectives on HCC treatment. Tumor immunotherapy and targeted therapy are currently hot topics in clinical research; however, there are still many difficulties to overcome in applying these approaches to solid tumors. This article reviews in-depth the composition and mechanical characteristics of the tumor microenvironment in HCC, its impact on immune cells, and potential immune and targeted therapies for HCC and other solid tumors. Encouraging results from various drug studies targeting these novel aspects have been reported. With the in-depth study of mechanical signaling, the concept of mechanical immune engineering has emerged, and it has been found that the hardness of tumor cells themselves can be altered and the mechanism of mechanical force regulation of immune cells can be utilized for tumor therapy. These concepts provide valuable experience for the next generation of tumor immunotherapy. However, the microenvironment of solid tumors is complex, variable and individualized, and many questions remain to be answered about the link between the mechanistic microenvironment and tumors; a more in-depth exploration of this topic could help to provide new ideas for HCC treatment.

## Figures and Tables

**Table 1:** Overview of drugs or methods targeting ECM

Drugs/methods	Target	Clinical Diseases	Reference/NCT Number
4MU	HA	HCC	113
HA-mediated Fe3O4 Nanocubes	EMT process markers	HCC	114
PEGPH20+Avelumab	HA+PD-L1	Pancreatic cancer	116 NCT03481920
Anti-VEGF therapy in combination with Chemotherapy	HA	mCRC	115
BAPN	LOX	Liver fibrosis	117
Losartan	LOX	Lung cancer	118
PXS-5153A	LOXL2/LOXL3	Liver fibrosis	119
Simtuzumab	LOXL2	Liver fibrosis	120-122, NCT01672866 NCT01672879

4MU: 4-methylumbelliferone; BAPN: β-aminopropionitrile; HA: Hyaluronic acid; mCRC: Metastatic colorectal cancer; LOX: Lysyl oxidase

**Table 2:** Overview of drugs or methods targeting CAF

Target	Drugs/methods	Clinical diseases	Reference
Target CAF subsets	Target α-SMA-positive or FAP-positive CAFs	Pancreas cancer; Lung cancer	123-125
	Cancer vaccine against FAP antigen	B16 melanoma model	126, 127
Normalize activated CAF and tumor mesenchyme	RA	PDAC	128, 129
	ATRA	PDAC	129
	VDR ligand calcipotriol	PDAC	130
	Halofuginone	PDAC	131
	Losartan	Breast and pancreatic cancer models	132

CAF: Cancer-associated fibroblasts; RA: Retinoic acid; ATRA: All-trans retinoic acid; VDR: Vitamin D receptor; PDAC: Pancreatic ductal adenocarcinoma; SCC: Squamous cell carcinoma

**Table 3:** Overview of drugs or methods targeting mechanosensors or mechanotransducers

Target	Drugs/methods	Clinical Diseases	Status	Reference/ NCT Number
Integrin α6β1	CD151 mAb 9B	HCC	Preclinical	136
YAP	Verteporfin	HCC	Preclinical	137
TRPV4	GL-V9	HCC	Preclinical	138
Rho/ROCK	Fasudil	HCC	Preclinical	139
Integrin avβ3	Synstatin	HCC	Preclinical	140
	ProAgio	Solid Tumor Malignancies	Recruiting; Phase 1	NCT05085548
	Etaracizumabs	Irinotecan-Refractor Advanced Colorectal Cancer	Completed; Phase 2	NCT00284817
FAK	AMP945 in Combination with Nab-paclitaxel and Gemcitabine	Pancreatic Cancer	Recruiting; Phase 1	NCT05355298
	VS-4718	Pancreatic Cancer	Terminated; Phase 1	NCT02651727
	Defactinib Combined with Pembrolizumab and Gemcitabine	Pancreatic Cancer	Completed; Phase 1	NCT02546531
Piezo1	Zuogui pill	Breast cancer bone metastasis	Preclinical	141

HCC: Hepatocellular carcinoma

## Abbreviations

HCC: hepatocellular carcinoma
TRP: transient receptor potential
TME: tumor microenvironment
ECM: extracellular matrix
PDAC: pancreatic ductal adenocarcinoma
HSCs: hepatic stellate cells
kPa: kilopascals
FIB-4: Fibrosis-4
IFP: interstitial fluid pressure
EMT: epithelial-mesenchymal transition
YAP: yes-associated protein
CSCs: cancer stem cells
VEGF: vascular endothelial growth factor
CAFs: cancer-associated fibroblasts
TAMs: tumor-associated macrophages
TIB: tumor immune barrier
DCs: dendritic cells
TANs: Tumor-associated neutrophils
TAN1: anti-tumor phenotype TANs
TAN2: pro-tumor phenotype TANs
NETs: neutrophil extracellular traps
HA: hyaluronic acid
mCRC: metastatic colorectal cancer
LOX: lysyl oxidase
CAR: Chimeric antigen receptor
